# Editorial: Biofilms and the “One Health” Concept: Human, Animals, and the Environment Depending on Community Life

**DOI:** 10.3389/fmicb.2022.869411

**Published:** 2022-03-15

**Authors:** Virgínia Farias Alves, Virginie Oxaran, Elaine C. P. De Martinis

**Affiliations:** ^1^Faculdade de Farmácia, Universidade Federal de Goiás, Goiânia, Brazil; ^2^Past Affiliated to the University of Texas at El Paso, El Paso, TX, United States; ^3^Faculdade de Ciências Farmacêuticas de Ribeirão Preto, Universidade de São Paulo, Ribeirão Preto, Brazil

**Keywords:** mixed species biofilms, one-health, omics, microniche, antimicrobials

Microorganisms appear very early in the timeline of our planet, millions of years before the first vertebrates. Indeed, from an evolutionary point of view, bacteria have had a long time to evolve and to adapt to diverse niches, compared to higher organisms. So it is not surprising that life on Earth is dependent on the tenuous equilibrium among humans, animals, and the environment–as postulated by the One Health concept. In this scenario, microorganisms are very important, and they can be either beneficial or detrimental, i.e., they play key roles in human and animal wellbeing or disease, in food production and spoilage, as well as in many industrial and wastewater treatment processes.

Considering the selective pressure from many harsh environments, microbial survival is highly favored by the formation of communities known as biofilms, where surface-associated microorganisms are encased in a protective extracellular polymeric matrix (EPS) containing open water channels. Biofilm development is a complex and dynamic process with several steps that include cell-surface attachment, EPS production, and population expansion. Microbial adhesion strategies are one of the main determinants for the development of biofilm-associated infections in clinics and biofouling in industrial settings, as discussed in the mini-review by Jiang et al..

Interestingly, although it is common to study single-species biofilms *in vitro*, this is not usually the pattern observed in natural settings, where complex polymicrobial communities are found, indicating that both synergic and/or antagonistic microbial social interactions are important. Several mechanisms that contribute to this interspecies networking are presented by Luo et al., helping to improve the current understanding of microbial cross-communication in natural environments, and shedding light on future strategies to counteract the harmful effects of biofilms. Corroborating the rationale that there is an interactive complexity among the members of biofilms, Burman and Bengtsson-Palme demonstrated that the use of model microbial consortia can add a lot of knowledge on what drives interactions in biofilms. In their study with a THOR model community (constituted of *Pseudomonas koreensis, Flavobacterium johnsoniae*, and *Bacillus cereus*), they observed that the amount of biofilm produced by THOR was strongly influenced by temperature changes, which suggests the existence of a temperature at which maximum community interaction occurs. Demonstrating such an effect is important for predicting global bacterial behavior, since temperature changes are likely to affect the interplay among all natural microbial communities, which can be reflected in the stability of an entire ecosystem.

On the other hand, to control biofilms formed by unwanted organisms, it is important to understand attachment mechanisms, since it is always easier to get rid of biofilms in early stages than attacking mature microbial communities. In this sense, Doghri et al. studied the effectiveness of natural substances (cell-free supernatants of coagulase-negative staphylococci and tomatidine, a steroid isolated from tomato plant) and synthetic compounds (zinc chloride and EDTA) to prevent biofilm formation by the foodborne pathogen *Listeria monocytogenes*. They demonstrated that the prevention of surface attachment by *L. monocytogenes* was associated with a disruption of bacterial motility, and suggested that flagellum-mediated motility represents a promising molecular target to avoid listerial persistence in food processing plants.

Moreover, the molecular characterization of different isolates of foodborne bacterial pathogens may indicate if there are strain-specific factors that contribute to their survival and persistence in food facilities and products. A study by Melo et al., based on the genetic analysis of *Salmonella* Heidelberg from Brazilian poultry, helped in explaining why this serovar has emerged as an important global threat, since all the strains displayed important virulence markers. According to Melo et al., among the strains studied there was a 70% prevalence of *luxS*, which is an important gene linked to quorum sensing and biofilm formation. Besides, two of the *Salmonella* Heidelberg strains presented more than 100 virulence genes, and both were resistant to 24 different classes of antimicrobial drugs. On top of these worrisome findings, it was shown that *Salmonella* Heidelberg strains from poultry presented the ability to form biofilms, and it is well known that sessile microorganisms may be even more tolerant to antimicrobials in comparison with planktonic cells.

From another point of view, the study of biofilm formation can also be relevant in clinics, with implications for the human host metabolism. In fact, the advent of “omic tools” has been crucial to decipher the complex diversity and the metabolic and functional dynamics of the gut microbiota, which is able to synthesize many bioactive compounds involved with the maintenance of host homeostasis. Zhang et al. studied the sex-specific differences in the association between gut microbiota and end-stage renal illness using biological indices and high-throughput sequencing of the 16S rRNA gene. Despite the sex-specific differences observed, the study results showed important correlations of gut microbiota and physiological/biochemical markers, concluding that the regulation of intestinal microbiota can contribute to both the prevention and treatment of end-stage renal diseases.

Altogether, this collection of articles shows that the investigation of biofilms remains a challenging field of research. Further studies on the mechanisms that govern their formation are still needed. Especially those based on novel molecular and advanced imaging techniques, to aid in the proposal of strategies to control biofilms of importance for promoting human life.

## Author Contributions

VA and ED: drafting of the manuscript. VA, VO, and ED: critical revision of the manuscript for important intellectual content. All authors contributed to the article and approved the submitted version.

## Conflict of Interest

The authors declare that the research was conducted in the absence of any commercial or financial relationships that could be construed as a potential conflict of interest.

## Publisher's Note

All claims expressed in this article are solely those of the authors and do not necessarily represent those of their affiliated organizations, or those of the publisher, the editors and the reviewers. Any product that may be evaluated in this article, or claim that may be made by its manufacturer, is not guaranteed or endorsed by the publisher.

